# On the Locus of L2 Lexical Fuzziness: Insights From L1 Spoken Word Recognition and Novel Word Learning

**DOI:** 10.3389/fpsyg.2021.689052

**Published:** 2021-07-08

**Authors:** Efthymia C. Kapnoula

**Affiliations:** Basque Center on Cognition, Brain and Language, San Sebastián, Spain

**Keywords:** word learning, fuzzy lexicon, mental lexicon, lexical representation, lexical representation and processing

## Abstract

The examination of how words are learned can offer valuable insights into the nature of lexical representations. For example, a common assessment of novel word learning is based on its ability to interfere with other words; given that words are known to compete with each other (Luce and Pisoni, 1998; Dahan et al., 2001), we can use the capacity of a novel word to interfere with the activation of other lexical representations as a measure of the degree to which it is integrated into the mental lexicon (Leach and Samuel, 2007). This measure allows us to assess novel word learning in L1 or L2, but also the degree to which representations from the two lexica interact with each other (Marian and Spivey, 2003). Despite the somewhat independent lines of research on L1 and L2 word learning, common patterns emerge across the two literatures (Lindsay and Gaskell, 2010; Palma and Titone, 2020). In both cases, lexicalization appears to follow a similar trajectory. In L1, newly encoded words often fail at first to engage in competition with known words, but they do so later, after they have been better integrated into the mental lexicon (Gaskell and Dumay, 2003; Dumay and Gaskell, 2012; Bakker et al., 2014). Similarly, L2 words generally have a facilitatory effect, which can, however, become inhibitory in the case of more robust (high-frequency) lexical representations. Despite the similar pattern, L1 lexicalization is described in terms of inter-lexical connections (Leach and Samuel, 2007), leading to more automatic processing (McMurray et al., 2016); whereas in L2 word learning, lack of lexical inhibition is attributed to less robust (i.e., fuzzy) L2 lexical representations. Here, I point to these similarities and I use them to argue that a common mechanism may underlie similar patterns across the two literatures.

## A Theoretical Framework for Evaluating Lexicalization

**FIGURE 1:**
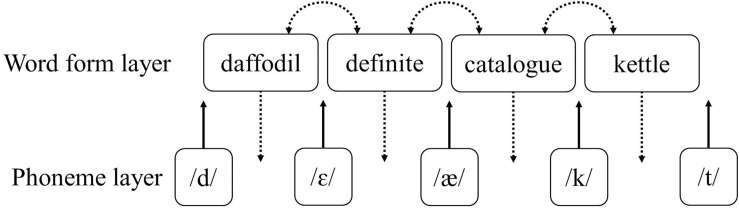
Visualization of lexical configuration versus lexical engagement properties in the context of spoken word recognition. Note that representations are depicted as localist nodes for ease of visualization, but no theoretical commitment is made regarding their nature.

Knowing a word means it is part of one’s mental lexicon. Thus, learning a new word requires integrating its representation in the mental lexicon in a way that allows it to be accessed (recognized and produced) in real time. According to Leach and Samuel (2007), this integration can be described as the acquisition of two lexical properties. *Lexical configuration* refers to the minimum amount of information required to “know” a word-form, which allows listeners to recognize it. This property consists of bottom-up pathways that map acoustic or phonological information to words (upward arrows in Figure 1). Then, *lexical engagement* refers to how a word interacts with other words (links between words in Figure 1), or lower level representations (top-down connections in Figure 1).

Indeed, there is robust evidence for lexical engagement, both in the form of words inhibiting each other (Luce and Pisoni, 1998; Vitevitch and Luce, 1998; Dahan et al., 2001), and in the form of top-down flow of information affecting perception of speech sounds in real-time (Magnuson et al., 2003; Samuel and Pitt, 2003; Luthra et al., 2021) and over the course of learning (Norris et al., 2003; Kraljic and Samuel, 2006).

Within this framework, we can use these two lexical properties to assess novel word learning. That is, we know that real words can affect the perception of speech sounds (Samuel and Kraljic, 2009; Luthra et al., 2021). For example, Warren (1970) showed that if we take a word (e.g., “legislature”) and replace one speech sound (e.g., the /s/) with a cough sound, listeners report that the original sound is there. This is known as *phonemic restoration* (see also Samuel, 1996). Another example is *perceptual learning* (Norris et al., 2003). Here we replace a speech sound with an ambiguous sound (e.g., we replace the /s/ in “personal” with a sound in-between /s/ and /∫/). If participants are exposed to many words like this, they learn to perceive the ambiguous sound as an /s/. In both cases, the effect can only be driven by real words. This means that, if a novel word can drive such top-down effects, this can be taken as evidence for lexicalization. Indeed, Leach and Samuel (2007) used this assessment to examine how several factors affect word learning. Participants learned a number of novel words and then it was assessed how well those items were integrated into the lexicon by measuring their ability to affect the perception of speech sounds (by driving phonemic restoration and perceptual learning). New words acted as real words in driving these effects, but only in some cases, depending on the details of the training procedure. Thus, this kind of lexicality test can help us assess which training works better and offer insights into the process of lexicalization.

Following a similar rationale, since known words compete with each other, we can use the capacity of a novel word to interfere with other lexical representations as a measure of the degree to which it is integrated into the mental lexicon. For example, Gaskell and Dumay (2003) examined the conditions under which newly learned words form inhibitory links with known words. Participants learned new words that overlapped with real L1 words (e.g., novel word: “cathudruke” overlapping with known word: “cathedral”). The results showed that newly learned words did not interfere with the recognition of their known-word competitors immediately after learning, but they did so after 3 days of training (see also Dumay and Gaskell, 2007; Bakker et al., 2014; Kapnoula et al., 2015; Kapnoula and McMurray, 2016a, for similar use of lexical competition as evidence for lexicalization). In addition, a reversal of the effect has been observed at the earliest stages of learning, with new words facilitating the recognition of similar-sounding words (Dumay and Gaskell, 2012). Thus, a shift from facilitation to inhibition is thought to reflect lexicalization.

These results demonstrate how different training parameters can lead to different outcomes in terms of how well a new word is integrated into the mental lexicon. In turn, the degree of lexical integration has implications for real-time recognition; well-integrated words are better (i.e., more automatically) recognized (for a review on the relationship between lexical integration and recognition automaticity, see McMurray et al., 2016). Critically, differences in how word recognition unfolds in real time are observed well beyond the initial stages of learning. For example, divergence from typical L1 spoken word recognition has been reported for individuals with specific language impairment (McMurray et al., 2010), developmental language disorder (McMurray et al., 2019b), and cochlear-implant users (McMurray et al., 2019a), while even within typically developing/hearing individuals, the way in which spoken words are recognized in real time changes over development (Rigler et al., 2015). These results, suggest that automaticity of word recognition can vary even amongst well-known, familiar words. In line with this idea, a study by Kapnoula and McMurray (2016b) found that the real-time dynamics of L1 word recognition are malleable. Participants were exposed to familiar words and each one was assigned to one of two experimental groups; in the high-competition group, pairs of similar-sounding familiar words (e.g., “net” and “neck”) were presented close together (temporally and/or spatially) in a manner that required participants to resolve the competition between them. In contrast, in the low-competition group, co-activation of words in each pair was minimized. After a 40-min exposure phase, the authors used a visual world paradigm task to track the time-course of lexical competition between words in each pair. They found that only participants in the high-competition group were able to fully suppress the activation of the competitor word. Moreover, computer simulations (using jTRACE; Strauss et al., 2007) pointed to increased inter-lexical inhibition as the parameter that helped participants in the high-competition group better suppress competitors.

Based on the studies presented above, we can conclude the following: First, it is broadly accepted that lexicality (i.e., lexical status) can be defined on the basis of how well a word is interlinked with other representations (e.g., other words) and that assessing the formation of these links can help us evaluate the degree to which a novel word has been learned. Second, such links are malleable, even for well-known L1 words, in the sense that they can be fine-tuned, possibly to accommodate short- and long-term demands of the language comprehension system. How is this framework relevant to L2 word learning?

## Evaluating Lexicalization in L2

To address this question, one must take into account the additional factor of phonological differences between L1 and L2; that is, non-native listeners often have to learn to distinguish between words based on L2 phonological contrasts that do not exist in their native language (Cutler and Otake, 2004; Weber and Cutler, 2004). For example, Dutch listeners find it difficult to differentiate between the English phonemes /æ/ and /ε/, which means they likely activate both “definite” and “daffodil” when hearing /daef/. Indeed, using a cross-modal priming paradigm, Broersma and Cutler (2011) found that hearing /daef/ facilitated visual recognition of the word “definite” for Dutch, but not for native English listeners. This pattern of results is taken as evidence for *phantom activation* in L2 word recognition, which refers to the activation of irrelevant words that are treated by the system as lexical competitors due to phonological confusability. Interestingly though, this increased competitor activation does not necessarily lead to increased inhibition of the target word. Specifically, Broersma (2012) showed that for native English speakers, hearing “deficit” inhibited subsequent visual recognition of the word “daffodil,” but for Dutch speakers, its effect was *facilitatory* to the same degree as hearing the target word (“daffodil”).

This seemingly paradoxical pattern of results has been explained in terms of *fuzzy lexical representations* (Darcy et al., 2013; Cook and Gor, 2015; Gor, 2018; closely linked to the Lexical Quality hypothesis, Perfetti, 2007). According to this hypothesis, some L2 words are encoded in the mental lexicon in a phonolexically underdifferentiated (i.e., fuzzy) way (Gor, 2018). This happens when words include phonemes that belong to non-native contrasts, which makes them easily confusable for L2 listeners (e.g., the /æ/ and /ε/ contrast that does not exist in Dutch). In those cases, L2 listeners activate similar-sounding words – as is the case with “daffodil” being activated when L2 listeners hear “deficit.” Despite the increased number of competitors, their fuzziness makes them poor inhibitors. At the same time, the cumulative sublexical activation is facilitatory, leading to a facilitatory net effect. This pattern is also in line with work on L1 word recognition showing independent and opposite effects at the lexical and sublexical levels (Vitevitch and Luce, 1998, 1999).

Indeed, there is growing support for the idea that L2 lexical representations can be fuzzily encoded due to perceptual confusability at the phoneme level, with the key finding consisting in non-native facilitation in priming tasks (Ota et al., 2009; Gor et al., 2010; Cook and Gor, 2015; Cook et al., 2016; Gor, 2018; Gor and Cook, 2020). Moreover, L1 phonology appears to be relevant, even when processing takes place in the visual modality (Ota et al., 2009) – a finding that offers support for the idea that L2 lexical representations are shaped by L1 phonology. Lastly, this effect is more robust for less familiar/low-frequency words. In contrast, when L2 prime words are well known (i.e., highly familiar and/or frequent), they seem to drive an inhibitory effect, similar to that observed in native speakers, a modulation that has been attributed to decreased lexical fuzziness of high-frequency primes (Cook and Gor, 2015; Gor and Cook, 2020).

## Fuzzy Representations and Fuzzy Connections

Bringing the two lines of work together, we can think of how they fit together and how L2 word learning effects such as phantom activation can be explained within the theoretical framework described earlier.

First, there is a striking similarity between the two literatures; in both cases, robust lexicalization is manifested as an inhibitory effect (see also Marian and Spivey, 2003; Qiao and Forster, 2017). However, in L1 word learning, inhibition is attributed to robust inter-lexical connections (i.e., lexical engagement); whereas in L2 word learning, inhibition is thought to reflect higher-resolution/less fuzzy encoding. The two accounts differ in perhaps subtle, but theoretically important ways. In the first case, the quality of lexical representations is not solely defined by how well encoded they are (which would fit under the lexical configuration property); rather lexical quality is also determined by the links between a word and other representations and, thus may be better described as an emergent property of lexical processing. In that respect, the two accounts are not theoretically incompatible; indeed, a word could be both fuzzily encoded and weakly interconnected with other words. In fact, it makes sense that fuzzy lexical encoding would lead to weak inter-lexical connections (both for L2, but also less familiar L1 words); however, the reverse is not guaranteed–that is, weak inter-lexical connections are not necessarily due to fuzzy encoding.

Second, within a framework such as the one described for L1 word learning, words with ambiguous phonemes (as is the case with difficult, non-native contrasts) are expected to have connections of similar strength with both speech categories, because the categories themselves are not well separated. In that sense, phantom activation effects could again be attributed to less robust lexical engagement in the form of weak links between a word and its phonemes. That is, assuming a system such as the one shown in Figure 1, in which there is interactive activation between the lexical and sublexical layers (McClelland and Elman, 1986; Luthra et al., 2021), activation of “daffodil” should spread to both /æ/ and /ε/ categories for Dutch speakers, which in turn would strengthen activation of “definite.” Moreover, this sequence is expected to take place independent of the modality in which lexical activation is originally triggered (auditory or visual), making this account also compatible with cross-modal effects (Ota et al., 2009).

In sum, phantom activation, priming facilitation, and modulation of the priming effect by word frequency are all well-established effects in L2 word recognition and they are commonly attributed to the fuzzy encoding of L2 lexical representations. However, I argue that these effects can also be explained in terms of processing automaticity (McMurray et al., 2016) and lexical engagement (Leach and Samuel, 2007).

## Conclusion

My goal was to highlight similar patterns across the literatures on L1 and L2 word learning and contribute to the effort of drawing connections between them (Lindsay and Gaskell, 2010; Palma and Titone, 2020). In doing so, I focused on a set of behavioral effects that are commonly attributed to fuzzy L2 lexical representations and I briefly described how these effects could be explained within a different theoretical framework, taken from the L1 word learning literature. It is important to note that the two accounts are not mutually exclusive and that it would be difficult to experimentally disentangle between the two. Rather than arguing for one mechanism over another, the purpose of this piece is to urge both sides to work closer together, considering that a common mechanism may (at least partly) underlie similar patterns across the two literatures.

## Author Contributions

ECK confirms being solely responsible for the conception and drafting of this work and has approved the final manuscript for publication.

## Conflict of Interest

The author declares that the research was conducted in the absence of any commercial or financial relationships that could be construed as a potential conflict of interest.
